# Intraocular inflammation in a case of bee sting injury

**DOI:** 10.3205/oc000084

**Published:** 2018-02-22

**Authors:** Ekta Rishi, Pukhraj Rishi

**Affiliations:** 1 Shri Bhagwan Mahavir Vitreoretinal Services, Sankara Nethralaya, Chennai, India

**Keywords:** vitritis, uveitis, bee sting, pupil, choroidal detachment

## Abstract

A19-year-old man presented with decreased vision in the right eye following a bee sting injury, ten days back. Examination revealed conjunctival hyperemia at the site of the sting, anterior uveitis, vitritis, mild disc hyperemia, ocular hypotony, and striae at macula. Treatment was initiated elsewhere with topical antibiotics, steroid, and cycloplegic. Systemic steroids were added. Three weeks later, vitiritis resolved, intraocular pressure became normal and the vision improved from 20/60 to 20/20. However, a chorioretinal atrophy in the vicinity of the sting site was noted. Global electroretinogram (ERG) revealed reduced scotopic responses and depressed oscillatory potentials; even though the photopic response was normal. Multifocal ERG and microperimetry were normal. Two months later, after discontinuation of medication, a traumatic mydriasis with a sluggish pupillary reaction was noted. Sixteen months later, the fundus remained stable. This is a rare case report of a bee sting injury leading to anterior uveitis, vitritis, and cilio-choroidal detachment, mimicking endophthalmitis. Timely recognition of the cause of the intraocular inflammation and appropriate treatment led to optimal recovery.

## Introduction

Ocular injuries can occur following bites by bees, wasps, and ants which belong to the group hymenoptera [1]. The effects are both local and systemic, and are mainly due to the toxins elaborated by the sting. So far, the eye-related injuries reported are conjunctivitis, corneal infiltrates [1], [2], cataract formation, iritis, hyphaema, lens subluxation, and optic nerve damage [3], [4], [5], [6]. Corneal injury is the most commonly reported ocular injury. Recently, ischaemic optic neuropathy and cilio-choroidal detachment [7] have also been reported. Hereby, we report an unusual case of vitritis following a bee sting injury to the eye, hitherto unreported (PubMed search). 

## Case description

**Figure 1:**
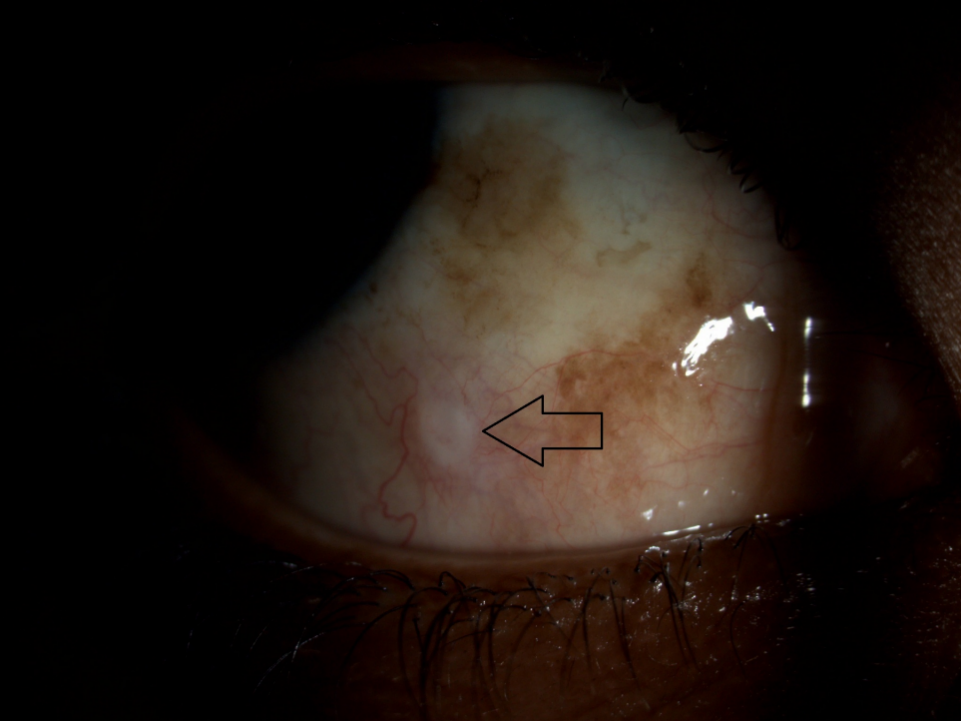
At presentation. External photograph of the right eye reveals conjunctival congestion in the inferonal quadrant of the eye with a prominent area of scleral protuberance (arrow) signifying the site of the bee sting injury.

**Figure 2:**
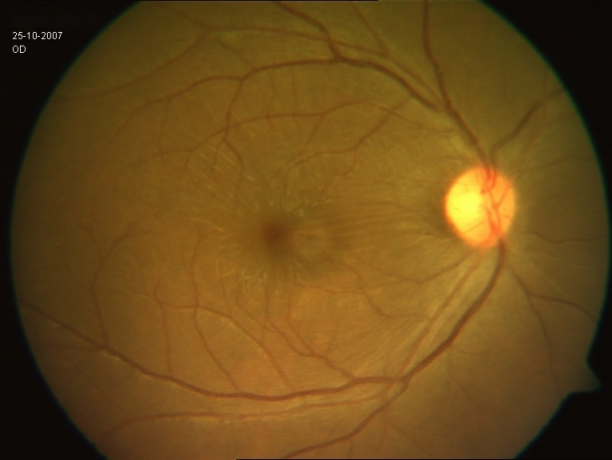
Color fundus photograph of the eye at presentation. Internal limiting membrane (ILM) folds over the macula and mild disc hyperemia are noteworthy.

**Figure 3:**
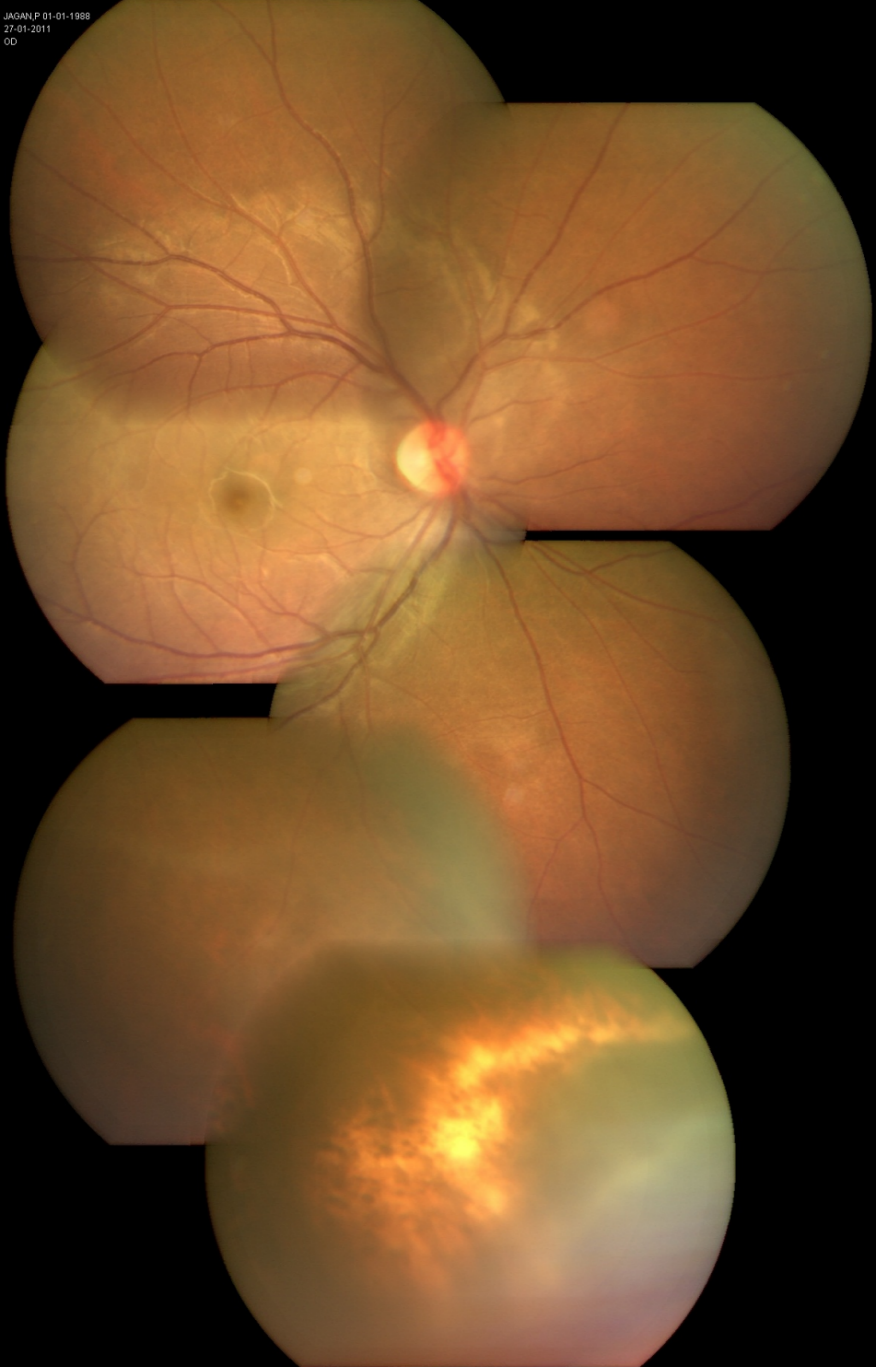
Three weeks after presentation. Color fundus montage of the right eye reveals minimal media haze overlying the affected quadrant with an area of chorioretinal pigmentation.

**Figure 4:**
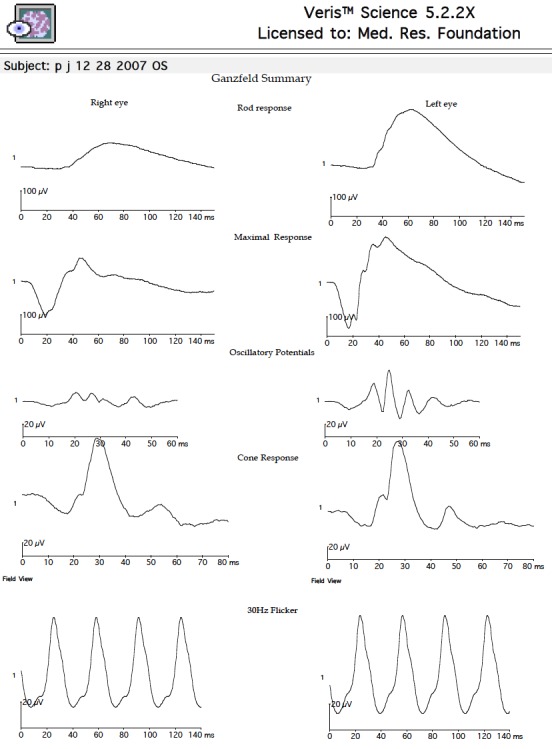
Global ERG reveals reduced scotopic responses and depressed oscillatory potential in the right eye. Photopic response was normal.

**Figure 5:**
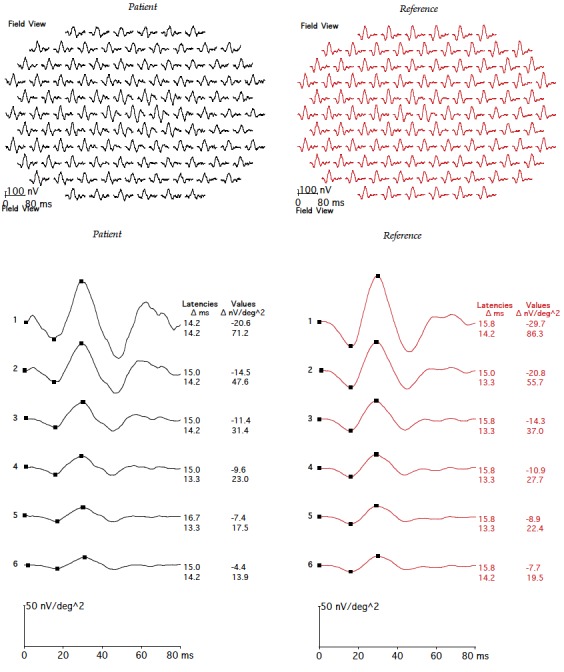
Multifocal ERG (trace array) reveals preserved macular function in the right eye.

**Figure 6:**
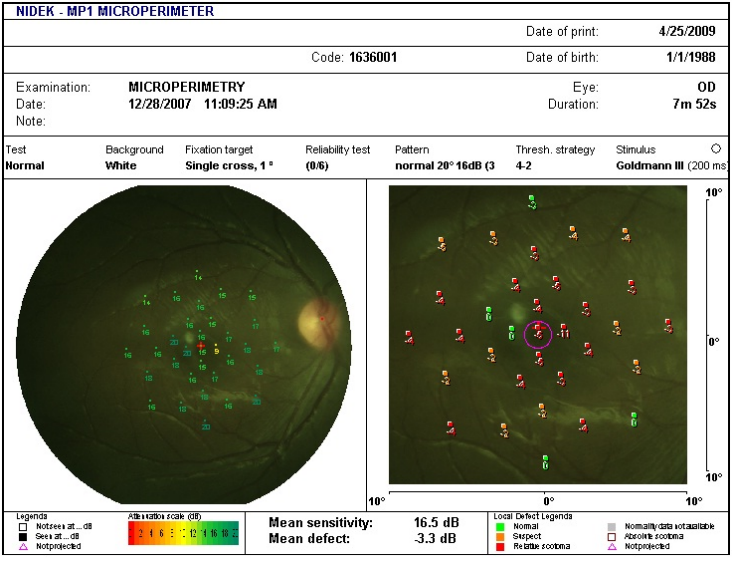
Macular perimetry reveals normal retinal sensitivity values with absence of scotoma.

A 19-year-old man presented to our emergency services with a ten-day history of decreased vision in the right eye following a bee sting injury. The patient had been treated by a local Ophthalmologist who initiated treatment with topical antibiotics, steroids, and cycloplegic agents. At presentation, best corrected visual activity (BCVA) was 20/60 in the right eye and 20/20 in the left. Examination revealed a localised area of conjunctival congestion and sloughing, inferonasal to limbus indicating the site of the sting injury (Figure 1 (Fig. 1)). Examination of the right eye revealed a pharmacologically dilated pupil, clear cornea and lens, 1+ cell in the anterior chamber, and 3+ cells in the vitreous cavity. Intraocular pressure (Goldman applanation) was 6 mmHg in the right eye, and 16 mmHg

in the left eye. Fundus examination revealed mild vitreous haze, mild disc hyperemia, and retinal striae at the macula (Figure 2 (Fig. 2)). The retinal details could not be seen clearly in the inferonasal fundus quadrant. The treatment already initiated elsewhere was continued and systemic steroids (oral Prednisolone 1 mg/kg) were added for the shallow, localized choroidal detachment in the affected quadrant. The patient was periodically followed. Three weeks after presentation, the pupil was pharmacologically dilated, vitritis dramatically reduced, and vision improved to 20/20. The conjunctival congestion was reduced. IOP was 18 mmHg. Fundus examination revealed minimal vitritis overlying the affected quadrant with an underlying area of chorioretinal pigmentation (Figure 3 (Fig. 3)). Optic disc hyperaemia was resolved. The retina was attached throughout and no obvious retinal break could be made out. Global electroretinogram (ERG) revealed reduced scotopic response and depressed oscillatory potentials in the right eye (Figure 4 (Fig. 4)), even though the photopic response amplitude was normal. Additionally, the trace array on multifocal ERG revealed intact ring responses (Figure 5 (Fig. 5)). Microperimetry (Figure 6 (Fig. 6)) revealed a normal retinal sensitivity without any scotoma. Clinical interpretation suggested a diffuse retinal damage to the outer retina (reduced scotopic response) and preserved inner retinal function (normal photopic response) with sparing of macular function (normal trace array on mfERG). Two months later, after discontinuation of all medications, a traumatic mydriasis with sluggish pupillary reaction to direct light reflex was noted. Fundus examination revealed the retina attached throughout with localised chorioretinal pigmentary changes in the affected quadrant and overlying vitreous condensation. The patient was periodically followed up and the pupillary size and reaction improved with time. At the last follow-up, 16 months following presentation, the fundus remained stable. The pupil was sluggishly reactive to direct light reflex and RAPD was noted to be absent. 

## Discussion

Bee sting injuries can lead to both local reactions like erythema at the site of the sting and systemic reactions like severe anaphylaxis, demyelination, and shock [1]. This occurs due to the toxic and immunologic response to a mixture of mediators like histamine, dopamine, polypeptide toxins, enzymes like phospholipase and hyaluronidase present in the bee venom. The reactions generally occur within minutes but can be delayed, too. Ocular changes have been reported involving the cornea [1], [2] and the optic nerve [3], [4], [5], [6], [7].

Ocular changes involving the retina [8] have been reported following a wasp sting but not a bee sting injury. Our patient presented with conjunctival hyperemia at the site of the sting, anterior uveitis, vitritis, mild disc hyperaemia, and retinal striae at the macula (pupil could not be assessed because of mydriatic use at that time). The histamine present in the bee sting leads to increase in the capillary permeability and can be the cause for the conjunctival hyperaemia at the site of the sting as well as the presence of cells in the anterior chamber. It is possible that the chemicals present in the bee sting might have entered the vitreous cavity leading to an area of localised vitritis. There has been a single report of ciliochoroidal detachment following a bee sting injury [7]. The initial intraocular pressure of our patient measured with Goldmann applanation tonometry was low; this could be due to hypotony caused by an abnormal accumulation of serous fluid in the choroid which might not have been clinically evident because of the overlying vitritis. Our patient had mild disc hyperaemia but the microperimetry reports were normal. Maltzmann et al. reviewed seven cases of optic neuropathy following a bee sting injury and found that optic disc edema, hyperaemia, and small disc hemorrhages were consistent findings in five patients [7]. Perimetry reportedly revealed central or caeco-central scotomas in all but the mildest case, which demonstrated only an enlarged blind spot in the affected eye. Relative afferent pupillary defects (RAPD) were reported in two cases. Our patient developed traumatic mydriasis and a sluggish reaction to light, detected after topical medications were discontinued. This could be due to a neurotoxic effect of the toxins. Our patient developed few retinal striae at the macula; this could be due to the ocular hypotony. Only a single case involving the retina where the electroretinography recordings became unrecordable has been reported, and that too following a wasp sting injury [8]. Our patient was treated with systemic steroids and steroids, antibiotics and cycloplegic agents. Three weeks after initiating treatment, the vision improved to 20/20 and the cellular reaction in the anterior chamber and the vitreous cavity reduced dramatically. The patient was followed-up for sixteen months. His vision was maintained at 20/20. The fundus appeared stable except for the stigmata of injury in the form of a localised area of chorioretinal atrophy in the infero-nasal quadrant. This is a rare case report of a bee sting injury leading to anterior uveitis, vitritis and ciliary detachment, mimicking endophthalmitis. Timely recognition of the cause of inflammation and appropriate treatment led to an optimal recovery. 

## Notes

### Competing interests

The authors declare that they have no competing interests.
